# Workplace social capital and self-rated health among nursery school teachers in Japan: a nationwide cross-sectional study

**DOI:** 10.3389/fpubh.2026.1718705

**Published:** 2026-03-16

**Authors:** Tomosa Mine, Satoshi Tsuboi, Yumi Yodogawa, Masahiro Imafuku, Hiroko Inokuma, Junko Minowa

**Affiliations:** 1 Department of Early Childhood Education and Care, Faculty of Education, Musashino University, Tokyo, Japan; 2 Department of Hygiene and Preventive Medicine, Fukushima Medical University, Fukushima, Japan; 3 Faculty of Education, Chiba University, Chiba, Japan; 4 Department of Early Childhood Care and Education, Komazawa Women's Junior College, Tokyo, Japan

**Keywords:** breaks, early childhood care and education, nursery school teachers, self-rated health, workplace social capital

## Abstract

**Introduction:**

Early Childhood Care and Education (ECCE) professionals, such as nursery school teachers in Japan, are essential for children's development but face substantial physical and emotional demands that may affect their health. In recent years, workplace social capital (WSC)—defined as trust, reciprocity, and cooperative relationships within the workplace—has been recognized as an important determinant of employee health. Although associations between WSC and health outcomes have been reported in several occupational groups, little is known about its role among ECCE professionals. This study investigated the relationship between WSC and self-rated health (SRH) among Japanese nursery school teachers caring for children under 3 years of age.

**Methods:**

We conducted a nationwide cross-sectional study from February to May 2024, targeting 5,000 randomly selected childcare facilities across Japan. Self-administered questionnaires collected data on demographics, work-hours–related conditions, WSC, and SRH. SRH was assessed using a single-item measure and dichotomized into “good” and “poor.” WSC was measured using the Japanese version of the Finnish Public Sector Study (FPSS) scale and categorized into four groups. Logistic regression models estimated crude and adjusted odds ratios (ORs) with 95% confidence intervals (CIs).

**Results:**

Of 2,470 valid responses, 10.4% reported poor SRH. Compared with the very high WSC group, participants in the very low WSC group had significantly higher odds of poor SRH (adjusted OR = 5.60; 95% CI: 3.24–9.70). This association remained significant after adjusting for work-hours–related conditions. Notably, additional adjustment for taking daily rest breaks attenuated the association (adjusted OR = 4.43; 95% CI: 2.40–7.00).

**Conclusion:**

This study provides the first nationwide evidence linking WSC with SRH among Japanese nursery school teachers. Lower WSC was strongly associated with poor SRH, independent of work-hours–related conditions. Supportive and cohesive workplace relationships may enhance perceived health and facilitate effective recovery from fatigue, underscoring their importance in ECCE settings.

## 1 Background

Healthy development of young children requires Early Childhood Care and Education (ECCE) professionals who are physically and mentally healthy, with an overall good wellbeing to lead high-quality childcare. In Japan, licensed ECCE professionals, such as nursery school teachers, play a central role in children's lives during the crucial developmental period from birth to age six, prior to mandatory education. Childcare for children from birth to 3 years is designed to integrate both care and education (1), and is provided by licensed nursery school teachers who obtain their qualifications through universities, junior colleges, vocational schools, or a national certification examination. To support children's healthy growth, their responsibilities extend beyond basic caregiving. They provide individualized emotional care and educational support, assist parents, and collaborate with colleagues and other professionals. In childcare for children under the age of three in particular, multiple teachers coordinate by exchanging information and allocating responsibilities. Effective collaboration in these settings is fostered through shared pedagogical values, mutual trust, and reciprocal support. This broad scope of responsibilities imposes significant physical and emotional demands on these professionals, often at the expense of ECCE professionals' own health and wellbeing. Previous studies have suggested that various factors contribute to the wellbeing of these professionals in ECCE settings, including burnout (2), stress (3), work environment (4), and overall health. However, large-scale epidemiological studies on wellbeing of the ECCE professionals remain limited internationally.

Self-rated health (SRH), a key indicator of overall health status, has been internationally validated (5) and is widely used in epidemiological and occupational health research (6, 7). In a general adult population, poor SRH has been shown to predict mortality (8) and is associated with factors such as age (9), lower socioeconomic status (10), unhealthy lifestyle behaviors (11), and physical and mental impairments (12). More specifically among teachers, poor SRH has been linked to physical complaints (e.g., musculoskeletal pain and voice disorders), mental health issues (e.g., depression and emotional exhaustion), low perceived professional efficacy, and increased risks of both absenteeism and presenteeism (13). We adapted SRH to assess the health and wellbeing of nursery school teachers in our large-scale surveys.

The field of ECCE is widely recognized as predominantly female (14). Female teachers, compared to their male counterparts, are more likely to experience high levels of emotional exhaustion and stress, particularly with respect to their interactions with colleagues in compulsory education (15). In recent years, workplace social capital (WSC), defined as a set of workplace resources encompassing trust, mutual support, shared values, and interactions within employee networks, has been recognized as an important work-related factor affecting employee health (16). Previous studies reported associations between WSC and a variety of health (17, 18) and organizational outcome (19). Although it has been hypothesized that WSC may be linked to SRH (20), few large-scale quantitative studies have investigated this relationship in the ECCE setting.

According to the Job Demands–Resources (JD-R) model, employees' wellbeing depends on the balance between job demands and resources (21). High job demands, such as long working hours and overtime, are associated with poorer health outcomes. Adequate recovery opportunities, including sufficient rest breaks and time off, are essential for maintaining physical and mental health. Job resources, such as supportive interpersonal relationships and social support at work, can mitigate the adverse effects of high job demands and insufficient recovery time, promoting wellbeing. In early childhood care and education settings, teachers often work long hours and perform emotionally and physically demanding work, relying on workplace relationships as an important source of support. Therefore, in addition to examining the association between WSC and SRH, we examined working time–related factors (overtime work, rest breaks, and regularly scheduled days off per month) and assessed whether workplace social capital might modify the association between these working environment factors and SRH.

Building on the previous research, this study examines the association between WSC and SRH using nationwide survey data from Japanese nursery school teachers caring for children from birth to 3 years. Our aim is to provide new insights into the social determinants of health in the ECCE sector, as well as to inform evidence-based strategies for supporting the wellbeing of ECCE professionals. To our knowledge, this is the first study in Japan to examine the association between WSC and SRH among nursery school teachers. Given the emotionally demanding and relationally intensive nature of ECCE work (22), it is important to investigate how WSC relates to the health and wellbeing of these professionals, in order to contribute to the further development of the ECCE field, as understanding its impact on teachers' health and wellbeing is crucial for advancing the ECCE fields.

## 2 Methods

### 2.1 Study design

This study examined the association between workplace social capital (WSC) and self-rated health (SRH) among nursery school teachers in Japan using a nationwide cross-sectional survey. The primary focus of the analysis was to estimate the association between WSC and SRH. We first fitted a base multivariable logistic regression model adjusted for demographic and employment-related background characteristics. To explore whether specific working-hours–related factors could account for the association, additional models were constructed in which work-specific factors related to working hours were added individually to the same base model. These factors included, regularly scheduled days off per month, taking daily rest breaks, overtime work hours at the workplace per week, Take-home work hours per week, and Total overtime work hours at the workplace and at home per week. Each working-hours–related variable was examined in a separate model rather than cumulatively to avoid overadjustment and multicollinearity among closely related working-hours measures.

### 2.2 Study population and data collection

We conducted a cross-sectional study. The sampling frame was based on the Welfare and Medical Service Network System's 2023 Children and Childcare Information Disclosure System (Kokode Search), an official government database providing information on childcare facilities (23). From this database, 5,000 public or private childcare facilities across Japan that provided care and education for children under 3 years of age were selected as the target population. Facilities were selected through simple random sampling, with consideration given to the national distribution by prefecture, facility type (nursery schools, certified ECEC centers, and small-scale childcare centers), and operating body (public, social welfare corporations, private companies, etc.). Self-administered questionnaires were mailed to the selected facilities and distributed to nursery teachers responsible for caring for children under the age of three. The survey was conducted from February to May 2024. A total of 2,727 responses were obtained from 998 facilities (response rate: 20.0%). Of these, 2,470 respondents with complete data on SRH, WSC, and all covariates were included in the final analysis (valid response rate: 90.6%).

### 2.3 Self-rated health (SRH)

SRH was assessed using a single-item question: “*How would you describe your overall state of health?”* (5). Responses were recorded on a four-point Likert scale (1 = very healthy to 4 = unhealthy). For analysis, responses were dichotomized into two categories: the good health group (combining *very healthy* and *somewhat healthy*) and the poor health group (combining *not very healthy* and *unhealthy*). This categorization is in line with previous Japanese population-based studies (24, 25) in which respondents reporting poorer health (e.g., “poor” or “not very good”) were classified as having poor SRH, while the remaining categories were grouped as better health.

### 2.4 Workplace social capital (WSC)

WSC was assessed using the Japanese version of the Finnish Public Sector Study (FPSS) scale. The reliability and validity of the original scale were established by Kouvonen et al. (26), and adapted for Japan by Kawachi Ichiro et al. (27). This scale has previously been applied to various occupational groups in Japan, including workers at research institutions (28) and junior high school teachers (29), supporting its applicability across occupational groups. Eight items rated on a four-point Likert scale (1 = strongly disagree to 4 = strongly agree) were used, capturing both vertical (e.g., relationships with supervisors) and horizontal (e.g., relationships among colleagues) dimensions of social capital. To reflect the ECCE context, references to “supervisor” were adapted to roles such as “principal” or “director,” where appropriate.

The eight WSC items were as follows:

1. We have a “we are together” attitude.
2. People feel understood and accepted by each other.
3. People keep each other informed about work-related issues in the work unit.
4. Members of the work unit build on each other's ideas in order to achieve the best possible outcome.
5. People in the work unit cooperate in order to help develop and apply new ideas.
6. We can trust our principal.
7. Our principal treats us with kindness and consideration.
8. Our principal shows concern for our rights as an employee.

A total score (ranging from 8 to 32) was calculated by summing responses to all items. The internal consistency of the scale was high (Cronbach's alpha = 0.88). Based on quartile distribution, participants were classified into four groups representing varying levels of WSC: very low, low, high, and very high. Due to a large number of tied values at the quartile cut-off points, the resulting group sizes were not equal.

### 2.5 Covariates

We included several covariates associated with SRH based on general evidence on SRH (2, 24, 30) and practical considerations relevant to the working conditions of nursery school teachers. Demographic characteristics, such as sex (female, male, others) and age (< 30, 30–39, 40–49, 50–59, ≥60), were included because they are consistently associated with SRH. Employment-related background variables were also considered, including employment status (full-time, part-time), type of education for qualification (junior college or vocational school, university, certification exam, or other licenses), and years of employment at the current nursery school (< 1, 1–2, 3–5, 6–10, 11–20, ≥21). These variables reflect differences in job security, career stage, and work experience, which may influence health outcomes. In this study, we explored associations between working conditions related to working hours and SRH among nursery school teachers. The regularly scheduled days off per month was categorized into three groups (< 6, 6–7, ≥8 days). Participants were also asked about taking daily rest breaks during working hours (sufficient, somewhat sufficient, insufficient, not at all). Weekly overtime work hours at workplace and take-home work hours were included. Overtime work at workplace was categorized as none, less than 5, or 5 h or more, while take-home work hours was categorized as none, less than 2, or 2 h or more.

### 2.6 Statistical analysis

SRH was dichotomized into two categories (“good” and “poor”) based on participants' responses. Descriptive statistics were calculated according to SRH category. To examine the association between WSC and SRH, multiple logistic regression analyses were conducted to estimate crude and adjusted odds ratios (ORs) with 95% confidence intervals (CIs), as this approach is appropriate for a dichotomous outcome and allows for straightforward interpretation of health risk.

WSC was treated as a categorical variable with four levels (very low, low, high, and very high), with the “very high” WSC group serving as the reference category. This categorization was used to enhance interpretability and to facilitate comparison across distinct levels of WSC that are more easily related to real-world workplace conditions, as well as to explore potential non-linear associations in this exploratory study.

Logistic regression analyses were conducted using a crude model and a multivariable model. The multivariable model was adjusted for sex, age category, employment status, type of education for qualification, and years at the current nursery school. Additional analyses were then performed by examining work-specific factors related to working hours, labeled as Model 1 through Model 5. Each working-hours-related variable was examined in a separate model by adding it individually to the same base model (the multivariable model), rather than cumulatively including multiple working-hours variables in a single model. These variables included regularly scheduled days off per month (Model 1), taking daily rest breaks during working hours (Model 2), overtime work hours at the workplace per week (Model 3), take-home work hours per week (Model 4), and total overtime work hours at the workplace and at home per week (Model 5). For trend analysis, logistic regression was repeated, with WSC categories assigned integer values, to examine whether there was a graded association between decreasing levels of WSC and the odds of poor SRH.

Additionally, for Models 1 through 5, potential interaction effects between WSC and each working-hours-related condition were assessed by including two-way interaction terms in the models.

All statistical analyses were conducted using Stata version 17 for Mac (StataCorp LLC, College Station, TX, USA).

## 3 Results

### 3.1 Study sample characteristics

Consistent with the sampling strategy, the distribution of facilities included in the final analysis by prefecture, facility type, and operating body was broadly comparable to the national distribution of childcare facilities in Japan. The proportions of nursery schools, certified ECEC centers, and small-scale childcare centers were similar to those reported at the national level (Supplementary Tables S1, S2). Among the participants included in the dataset, those who provided complete data (*N* = 2,470) and those who did not provide data on the study variables (*N* = 257) showed similar basic characteristics.

Descriptive statistics of the study population included in the final analysis are presented in Table 1. Overall, 2,213 participants (89.6%) reported good health, of whom 434 (19.6%; 17.5% of the total) identified as “very healthy” and 1,779 (80.4%; 72.0% of the total) as “somewhat healthy.” In contrast, 257 participants (10.4%) reported poor health, including 241 (93.8%; 9.8% of the total) who described themselves as “not very healthy” and 16 (6.2%; 0.7% of the total) who described themselves as “unhealthy.” The mean WSC score was 25.0 (SD = 3.0). Based on quartiles, participants were categorized into four WSC groups: very low (scores 11–23; *n* = 811, 32.8%), low (scores 24–25; *n* = 523, 21.2%), high (scores 26–28; *n* = 715, 29.0%), and very high (scores 29–32; *n* = 421, 17.0%).

Table 1Characteristics of the participants by self-rated health.

**Table:** 

**Variable**	Self-rated health						
	Total (*n* = 2,470)		Good (*n* = 2,213, 89.6%)		Poor (*n* = 257, 10.4)		***p*** **-Value**
	***n***	**%**	***n***	**%**	***n***	**%**	
**Self-rated health**							
Very healthy	434	17.5	434	19.6	0	0.0	
Somewhat healthy	1,779	72.0	1,779	80.4	0	0.0	
Not very healthy	241	9.8	0	0.0	241	93.8	
Unhealthy	16	0.7	0	0.0	16	6.2	
**Gender**							
Female	2,413	97.7	2,163	97.8	250	97.3	0.184
Male	55	2.2	49	2.2	6	2.3	
Others	2	0.1	1	0.0	1	0.4	
**Age**							
< 30	685	27.7	645	29.1	40	15.6	< 0.001
30–39	680	27.6	613	27.7	67	26.1	
40–49	617	25.0	527	23.8	90	35.0	
50–59	379	15.3	329	14.9	50	19.5	
60 ≤	109	4.4	99	4.5	10	3.9	
**Employment status**							
Full-time	2,381	96.4	2,129	96.2	252	98.1	0.132
Part-time	89	3.6	84	3.8	5	1.9	
**Education for qualification**							
Junior college/vocational school	1,861	75.3	1,649	74.5	212	82.5	0.032
University	357	14.5	334	15.1	23	9.0	
Certification exam	225	9.1	205	9.3	20	7.8	
Others	27	1.1	25	1.1	2	0.8	
**Years of employment at the current nursery school**							
< 1	346	14.0	307	13.9	39	15.2	0.538
1–2	466	18.9	421	19.0	45	17.5	
3–5	457	18.5	409	18.5	48	18.7	
6–10	614	24.9	555	25.1	59	23.0	
11–20	438	17.7	394	17.8	44	17.1	
21 ≤	149	6.0	127	5.7	22	8.6	
**Regularly scheduled days off per month**							
< 6 days	124	5.0	110	5.0	14	5.4	0.251
6–7 days	760	30.8	670	30.3	90	35.0	
8 days ≤	1,586	64.2	1,433	64.7	153	59.5	
**Taking daily rest breaks during working hours**							
Sufficiently	556	22.5	532	24.0	24	9.3	< 0.001
To some extent	1,043	42.2	949	42.9	94	36.6	
Not much	618	25.0	531	24.0	87	33.9	
Not at all	253	10.2	201	9.1	52	20.2	
**Overtime work hours at workplace per week**							
None	1,398	56.6	1,266	57.2	132	51.4	0.001
≤5	829	33.6	746	33.7	83	32.3	
>5	243	9.8	201	9.1	42	16.3	
**Take-home work hours per week**							
None	1,496	60.6	1,377	62.2	119	46.3	< 0.001
≦2	342	13.9	310	14.0	32	12.5	
>2	632	25.6	526	23.8	106	41.3	
**Total overtime work hours at work place and home per week**							
None	1,058	42.8	977	44.2	81	31.5	< 0.001
≤5	1,068	43.2	957	43.2	111	43.2	
>5	344	13.9	279	12.6	65	25.3

**Table:** 

**WSC (score)**	**Mean (±sd)**							
Very low (11–23)	21.6 (±1.8)	811	32.8	666	30.1	145	56.4	< 0.001
Low (24, 25)	24.5 (±0.5)	523	21.2	478	21.6	45	17.5	
High (26–28)	27.0 (±0.8)	715	29.0	663	30.0	52	20.2	
Very high (29–32)	29.0 (±0.1)	421	17.0	406	18.4	15		

Most participants were female (97.7%) and employed full-time (96.4%). Approximately half were in their 20s or 30s (< 30 years: *n* = 685, 27.7%, 30–39 years: *n* = 680, 27.6%). A majority (75.3%) obtained their nursery school teacher qualification through a 2-year junior college or vocational school. About half of the participants had worked at their current nursery school for less than 5 years. While 64.2% reported having eight or more regularly scheduled days off per month, only 22.5% reported being able to take sufficient rest breaks during working hours. Notably, more than one-third of participants reported taking inadequate rest breaks (“not much” and “not at all”). Approximately 60.0% reported engaging in overtime work, including both workplace and take-home work.

### 3.2 WSC and SRH

The association between the WSC groups and odds ratio of poor SRH are shown in Tables 2–6. Compared with very high WSC, the crude ORs for poor SRH were 2.12 (95% CI, 1.18–3.82) for high WSC, 2.54 (95% CI, 1.39–4.63) for low WSC, 5.89 (95% CI, 3.41–10.17) for very low WSC. After adjusting for sex, age, employment status categorized as full-time or part-time, years of employment at the current facility, and the type of educational institution attended for qualification acquisition (Multivariable Model), the association remained significant: OR = 2.09 (95% CI, 1.15–3.77) for high WSC, OR = 2.45 (95% CI: 1.34–4.48) for low WSC and 5.64 (95% CI: 3.25–9.76) for very low. Further adjustment for individual working conditions—regularly scheduled days off per month (Model 1), overtime work hours at the workplace (Model 3), take-home work hours (Model 4), and total combined overtime work hours (Model 5)—did not substantially alter the associations. However, when taking daily rest breaks was included in Model 2, the odds ratios were moderately attenuated. For example, the adjusted OR for poor SRH in the very low WSC group decreased from 5.64 (95% CI: 3.25–9.76) to 4.43 (95% CI: 2.40–7.00). When WSC was treated as an ordinal variable, a significant graded trend of increasing odds of poor SRH with decreasing WSC was observed across all models (*p* for trend < 0.05). No statistically significant interactions were observed.

**Table 2:** Association between WSC status and poor self-rated health: logistic regression model adjusted for regularly scheduled days off (Model 1).

**Variable**	***n*/*N***	**(%)**	Crude ORs (95% CIs)		Multivariable model ORs (95% CIs)^**a**^		Further adjusted by regularly scheduled days off ORs (95% CIs)	
**WSC status**								
Very high	15/421	(3.5)	Ref		Ref			
High	52/715	(7.3)	2.12	(1.18–3.82)	2.09	(1.15–3.77)	2.09	(1.16–3.78)
Low	45/523	(8.6)	2.54	(1.39–4.63)	2.45	(1.34–4.48)	2.45	(1.34–4.47)
Very low	145/811	(17.9)	5.89	(3.41–10.17)	5.64	(3.25–9.76)	5.60	(3.24–9.70)
OR per 1-level decrease in WSC status			1.75	(1.53–2.00)	1.72	(1.51–1.98)	1.71	(1.50–1.97)
*p* for trend			<0.05		<0.05		<0.05	
**Regularly scheduled days off**								
6>	–						Ref	
6–7	–						1.16	(0.87–1.55)
8 ≤	–						1.05	(0.57–1.91)

CI, confidence interval.

^a^Adjusted for sex, age category, employment status, education for qualification, and years of employment at the current nursery school.

**Table 3:** Association between WSC status and poor self-rated health: logistic regression model adjusted for taking daily rest breaks during working hours (Model 2).

**Variable**	***n*/*N***	**(%)**	Crude ORs (95% CIs)		Multivariable model ORs (95% CIs)^**a**^		Further adjusted by taking daily rest breaks during working hours ORs (95% CIs)	
**WSC status**								
Very high	15/421	(3.5)	Ref		Ref			
High	52/715	(7.3)	2.12	(1.18–3.82)	2.09	(1.15–3.77)	1.95	(1.07–3.52)
Low	45/523	(8.6)	2.54	(1.39–4.63)	2.45	(1.34–4.48)	2.03	(1.10–3.74)
Very low	145/811	(17.9)	5.89	(3.41–10.17)	5.64	(3.25–9.76)	4.43	(2.40–7.00)
OR per 1-level decrease in WSC status			1.75	(1.53–2.00)	1.72	(1.51–1.98)	1.59	(1.38–1.83)
*p* for trend			<0.05		<0.05		<0.05	
**Taking daily rest breaks during working hours**								
Sufficient	–						Ref	
Somewhat sufficient	–						1.75	(1.09–2.81)
Insufficient	–						2.51	(1.54–4.08)
Not at all	–						4.10	(2.40–7.00)

CI, confidence interval.

^a^Adjusted for sex, age category, employment status, education for qualification, and years of employment at the current nursery school.

**Table 4:** Association between WSC status and poor self-rated health: logistic regression model adjusted for overtime work hours at workplace per week (Model 3).

**Variable**	***n*/*N***	**(%)**	Crude ORs (95% CIs)		Multivariable model ORs (95% CIs)^**a**^		Further adjusted by overtime work hours at workplace per week ORs (95% CIs)	
**WSC status**								
Very high	15/421	(3.5)	Ref		Ref			
High	52/715	(7.3)	2.12	(1.18–3.82)	2.09	(1.15–3.77)	2.08	(1.15–3.76)
Low	45/523	(8.6)	2.54	(1.39–4.63)	2.45	(1.34–4.48)	2.40	(1.31–4.39)
Very low	145/811	(17.9)	5.89	(3.41–10.17)	5.64	(3.25–9.76)	5.46	(3.15–9.48)
OR per 1-level decrease in WSC status			1.75	(1.53–2.00)	1.72	(1.51–1.98)	1.70	(1.49–1.96)
*p* for trend			<0.05		<0.05		<0.05	
**Overtime work hours at workplace per week**								
None	–						Ref	
≤5	–						1.00	(0.74–1.34)
>5	–						1.71	(1.15–2.53)

CI, confidence interval.

^a^Adjusted for sex, age category, employment status, education for qualification, and years of employment at the current nursery school.

**Table 5:** Association between WSC status and poor self-rated health: logistic regression model adjusted for take-home work hours per week (Model 4).

**Variable**	***n*/*N***	**(%)**	Crude ORs (95% CIs)		Multivariable model ORs (95% CIs)^**a**^		Further adjusted by take-home work hours per week ORs (95% CIs)	
**WSC status**								
Very high	15/421	(3.5)	Ref		Ref		Ref	
High	52/715	(7.3)	2.12	(1.18–3.82)	2.09	(1.15–3.77)	2.04	(1.13–3.69)
Low	45/523	(8.6)	2.54	(1.39–4.63)	2.45	(1.34–4.48)	2.29	(1.21–4.19)
Very low	145/811	(17.9)	5.89	(3.41–10.17)	5.64	(3.25–9.76)	5.19	(3.00–9.01)
OR per 1-level decrease in WSC status			1.75	(1.53–2.00)	1.72	(1.51–1.98)	1.67	1.46–1.92
*p* for trend			<0.05		<0.05		<0.05	
**Take-home work hours per week**								
None	–						Ref	
≤2	–						1.01	0.71–1.65
>2	–						2.02	1.51–2.71

CI, confidence interval.

^a^Adjusted for sex, age category, employment status, education for qualification, and years of employment at the current nursery school.

**Table 6:** Association between WSC status and poor self-rated health: logistic regression model adjusted for total overtime hours at workplace and home per week (Model 5).

**Variable**	***n*/*N***	**%**	Crude ORs (95% CIs)		Multivariable model ORs (95% CIs)^**a**^		Further adjusted by total overtime hours at workplace and home per week ORs (95% CIs)	
**WSC status**								
Very high	15/421	(3.5)	Ref		Ref		Ref	
High	52/715	(7.3)	2.12	(1.18–3.82)	2.09	(1.15–3.77)	2.01	(1.13–3.72)
Low	45/523	(8.6)	2.54	(1.39–4.63)	2.45	(1.34–4.48)	2.32	(1.17–4.25)
Very low	145/811	(17.9)	5.89	(3.41–10.17)	5.64	(3.25–9.76)	5.21	(3.00–9.04)
OR per 1-level decrease in WSC status			1.75	(1.53–2.00)	1.72	(1.51–1.98)	1.67	(1.46–1.92)
*p* for trend			<0.05		<0.05		<0.05	
**Total overtime hours at workplace and home per week**								
None	–						Ref	
≤5	–						1.24	(0.91–1.67)
>5	–						2.37	(1.64–3.42)

CI, confidence interval.

^a^Adjusted for sex, age category, employment status, education for qualification, and years of employment at the current nursery school.

## 4 Discussion

This nationwide cross-sectional study investigated the relationship between WSC and SRH among Japanese nursery school teachers who care for children from birth to 3 years. The findings suggest that lower levels of WSC are significantly associated with an increased likelihood of poor SRH, independent of individual characteristics and work-hours-related factors. Notably, this association remained statistically significant, even after adjusting for key work-hours–related variables, such as regularly scheduled days off per month, taking daily rest breaks per week, and overtime work hours per week. Furthermore, when WSC was modeled as an ordinal variable, a significant graded trend was observed, with progressively lower WSC associated with higher odds of poor SRH across all models. This graded pattern suggests that the association between WSC and SRH followed a consistent gradient across WSC categories, rather than being driven only by extreme groups. This supports our research question by showing a graded association between WSC and SRH across successive ordered categories, rather than only between the lowest and highest categories.

In this study, WSC—defined as social resources such as trust, reciprocity, and cooperation within the workplace—was significantly associated with SRH, reflecting individuals' subjective assessments of their own health status. Participants were employed at randomly selected nursery schools across Japan, reflecting diversity in workplace environments. Despite this variation, a consistent association between WSC and SRH was observed, suggesting that the impact of WSC on perceived health may be generalizable across different nursery school settings. This result is consistent with findings from prior studies among nurses (30) and other human service professionals (31), where WSC has also been shown to impact health outcomes.

Nursery school teachers and nurses are typically female-dominated professions. In occupations related to nursing and childcare, the proportion of women often exceeds 90% (32). Consistent with this structural characteristic, the vast majority of participants in our study were women. In the present study, male participants accounted for 2.2% of the sample, which is lower than the approximately 6.6% reported in the 2024 Basic Survey on Wage Structure conducted by the Ministry of Health, Labor and Welfare (33). Although the proportion observed in this study does not correspond exactly to national figures, both indicate that the childcare workforce in Japan is overwhelmingly female-dominated. The lower proportion of male participants in the present study may be partly attributable to its focus on childcare workers primarily responsible for the care of children under 3 years of age, a subgroup in which female workers are more highly represented.

These professions involve emotionally and physically demanding work settings with significant responsibilities (34, 35), long and irregular working hours (36), and emotionally intense interpersonal demands (37). Prior studies on WSC in the nursing profession (38, 39) suggest that these findings could provide meaningful guidance in addressing health issues among ECCE professions. In addition, sensitivity analyses restricted to female participants yielded results consistent with the main analyses, suggesting that the observed associations were unlikely to be materially affected by the small proportion of male respondents.

In Japan, working conditions in the ECCE sector are a growing concern, including limited opportunities to take adequate breaks and frequent overtime work (40). These structural challenges have been consistently identified as widespread issues in the ECCE field, yet empirical studies examining their health impacts remain limited. In this context, our study investigated whether work-hours–related factors—such as regularly scheduled days off per month, taking daily rest breaks, and weekly overtime work hours (workplace and/or take-home work)—could account for the association between WSC and SRH. Notably, even after adjusting for each of these variables individually, the association between WSC and poor SRH remained statistically significant.

Importantly, however, one of the key findings was that adjusting for taking daily rest breaks resulted in the greatest attenuation of the association between low WSC and poor SRH. This indicates that rest breaks may play a particularly important role in this relationship. This pattern suggests that the ability to take rest breaks may reflect workplace social processes related to WSC, such as mutual support and coordination among teachers. Workplace climate and cultural norms further shape break-taking practices. Employees are often reluctant to take micro-breaks or self-initiated breaks because of concerns that their tasks for the day will not be completed, or worries about how such behavior may be perceived by supervisors and coworkers (41). In ECCE settings, it is likely that the feasibility of taking rest breaks similarly depends on supportive interpersonal relationships and cooperation among colleagues and supervisors.

Vieten et al. (42) reported that the frequency of skipped or interrupted breaks has a greater impact on employees' health than the duration of breaks. When interruptions are frequent, the total length of break time plays a relatively minor role in determining health outcomes. Moreover, it has been pointed out that physically leaving the work environment during breaks may be more effective for fatigue recovery than the duration of breaks itself (43). In Japan, many ECCE teachers—including licensed nursery school teachers and kindergarten teachers—take their breaks within the facility. It is not uncommon for facilities to lack formally designated rest periods, and depending on daily staffing arrangements, it is often difficult for staff to take breaks on their own initiative.

Consequently, teachers may find it difficult to physically distance themselves from their duties, which increases the likelihood of interruptions or skipped breaks and may limit effective recovery from fatigue, thereby potentially affecting SRH.

Taken together, these findings suggest that the attenuation observed after adjustment for daily rest breaks may indicate that break-taking practices represent a potential factor linking WSC to SRH. Workplaces with higher WSC may be characterized by greater mutual support, coordination, and flexibility in task sharing, which can enable employees to take needed rest breaks. In this sense, rest breaks may function not only as an individual behavior but also as an organizationally enabled practice that reflects underlying social and relational workplace resources in ECCE settings.

Although adjustment for overtime work and take-home work did not materially change the estimates in our models, prior research indicates that workload demands and time pressure can constrain opportunities to take rest breaks by reducing employees' ability to disengage from work (41). In ECCE settings, teachers often prioritize time with children and complete administrative and preparatory tasks outside paid working hours, shifting part of their workload into unpaid time (44). Qualitative studies from Australia and New Zealand also suggest that unpaid overtime is widespread among early childhood educators, although large-scale quantitative evidence remains limited (45, 46). Together, these findings suggest that break-taking practices may be partly shaped by broader workload conditions.

In the present study, 10.5% of participants reported poor SRH—a relatively low proportion. Compared with data from the general population of Japanese women, this rate appears slightly lower. For example, a previous study reported that 18.9% of full-time employed women aged 40–59 years in Japan had poor SRH (24). Similarly, a study of female healthcare university students in Japan, with a mean age of 19.4 years, found a poor SRH prevalence of 11.4% (47). Furthermore, a study of female apprentice kindergarten teachers aged 16–30 years in Germany reported a higher prevalence of poor SRH (20.7%) than that observed among participants in their 20s in our sample (15.6%) (48).

These differences may be partly explained by the age distribution of the participants. In our study, 27.7% were in their 20s and 27.5% in their 30s, meaning that over half of the participants were under the age of 40, indicating a relatively young sample. According to a national report by the Ministry of Health, Labor and Welfare, approximately 32.9% of childcare facility staff are under 30 and 25.6% are in 30s in Japan (49). These figures suggest that our sample is broadly representative of the national ECCE workforce in terms of age distribution.

SRH is known to be influenced by various factors, including age (9), the presence of chronic conditions (50), and health literacy (51). Among ECCE professionals, a high prevalence of occupational health issues—such as burnout (2) and musculoskeletal pain (e.g., low back pain) (52, 53)—has been reported. However, these conditions may not be fully captured by SRH alone. Therefore, while our findings offer important insights, they should be interpreted with caution. Future studies should adopt more comprehensive health indicators, including objective physical and mental health assessments to more accurately evaluate the overall health status of ECCE professionals.

## 5 Limitations and strengths

This study has several limitations. First, due to its cross-sectional design, conclusive connections between WSC and SRH cannot be established. Although a graded trend was observed across WSC levels, this pattern should be interpreted with caution because the cross-sectional design does not permit causal inference. Second, although prior research has shown associations between SRH and factors such as socioeconomic status (24, 54), lifestyle behaviors (e.g., tobacco use) (55, 56), and health literacy (51), information on participants' socioeconomic conditions (e.g., income and household financial status) and specific health-related behaviors was not collected in our study. This was primarily due to constraints related to the Japanese cultural context, including difficulties in collecting sensitive personal information such as socioeconomic status and educational background, as well as concerns about respondent burden that could negatively affect response rates. Consequently, we were unable to adjust for these potentially confounding variables, and residual confounding cannot be ruled out. Third, facility-level characteristics, such as center size and staff-to-child ratios, were not measured, limiting our ability to examine their influence on SRH.

Additionally, the response rate was approximately 20%. It is possible that those who participated had more time to complete the survey or were employed in more supportive environments characterized by favorable leadership and organizational culture. Indeed, respondents tended to report relatively high WSC scores, suggesting the presence of a ceiling effect. In addition, the study population consisted of nursery school teachers caring for children under 3 years of age, a work setting in which close coordination, mutual support, and effective communication among staff are particularly important to ensure child safety. This occupational characteristic may partly contribute to the relatively high overall levels of WSC observed in the present study. Furthermore, caring for infants and toddlers typically demands a high level of physical and mental wellbeing, which may partly explain why those working in this area tend to report better health. As a result, nursery school teachers with poorer health or those working in more demanding or unfavorable environments may have been less likely to participate in the survey. This may have introduced selection bias and potentially led to an underestimation of the true association. Consequently, the present findings may represent a conservative estimate of the health risks associated with unfavorable WSC, and the true impact in certain ECCE work environments with higher psychosocial and physical demands may be even greater.

Finally, although the WSC scale was adapted for use in the nursery school workforce, it was originally developed to assess WSC in the general workforce. Further research is needed to validate its contextual relevance for ECCE professionals.

Despite these limitations, the association between WSC and SRH remained statistically significant across all adjusted models, supporting the robustness of the findings. A notable strength of this study is its use of a nationwide sample of nursery school teachers employed in both public and private nursery school facilities across Japan. Given the limited research on the health of ECCE professionals in Japan, these findings contribute important empirical evidence to a critical yet underexplored area of occupational health.

In this study, SRH was assessed as a single indicator of overall health. However, health is a multidimensional concept that encompasses physical, mental, and social aspects. Future research should therefore examine the health of nursery school teachers using more comprehensive and multidimensional measures. Despite these limitations, quantitative studies on the health of early childhood educators remain scarce, both in Japan and internationally. Within this context, the present study offers meaningful insights into the discussion on occupational health in the ECCE sector.

## 6 Conclusion

This study examined the association between WSC and SRH among nursery school teachers responsible for children under the age of three across Japan. The results demonstrated that lower levels of WSC were significantly associated with poorer SRH, even after adjusting for factors such as age, gender, employment type, and work-related conditions. Notably, the difficulty in taking daily rest breaks appeared to partially attenuate this association, suggesting that not only structural work environments but also interpersonal relationships and support within the workplace play a critical role in health outcomes.

These findings highlight the importance of fostering trust, reciprocity, and cooperation in the workplace to support the health of ECCE professionals. Organizational strategies that promote supportive social relationships and ensure accessible, uninterrupted rest breaks may contribute to improved employee wellbeing.

Quantitative studies focusing on the health of ECCE professionals remain scarce, not only in Japan but also internationally. This study contributes valuable empirical evidence to this underexplored yet vital occupational group. Future research should include longitudinal studies to clarify the causal pathways through which WSC influences health, taking into account the unique work culture and practices of ECCE settings. Additionally, intervention studies aimed at improving workplace environments are warranted to further promote the health and wellbeing of ECCE professionals.

## Data Availability

The raw data supporting the conclusions of this article will be made available by the authors, without undue reservation.
