# Supplementary material for: Workplace social capital and self-rated health among nursery school teachers in Japan: a nationwide cross-sectional study
Source: Front Public Health. 2026 Mar 16;14:1718705. doi: 10.3389/fpubh.2026.1718705 (PMC13033703; doi:10.3389/fpubh.2026.1718705)
Supplement: Supplementary file 1 [file Table_1.docx]

**Supplementary Table**

**Table S1. Distribution of Facilities by Sampling Criteria: National, Shipped, Returned, and Analyzed Samples**

|  | National*  N, % | | Shipped  N, % | | Return  N, % | | Analyzed sample  N, % | |
| --- | --- | --- | --- | --- | --- | --- | --- | --- |
| Nursery school | 21755 | 58.3 | 3071 | 61.5 | 1520 | 55.7 | 1361 | 55.1 |
| ECEC center | 8880 | 23.8 | 1061 | 21.2 | 632 | 23.2 | 581 | 23.5 |
| Small-scale  childcare center | 5983 | 16.0 | 763 | 15.3 | 479 | 17.6 | 439 | 17.8 |
| others | 670 | 1.8 | 102 | 2.0 | 61 | 2.2 | 56 | 2.3 |
| missing | - | - | 5000 |  | 35 | 1.3 | 33 | 1.3 |
| Total | 37288*^1^ | 99.9 | 4997 | 100.0 | 2727 | 100.0 | 2470 | 100.0 |

**Table S2. Distribution of Operating Bodies by Sampling Criteria: National, Shipped, Returned, and Analyzed Samples**

|  | **National**  **N, %** | | **Shipped**  **N, %** | | **Return**  **N, %** | | **Analyzed sample**  **N, %** | |
| --- | --- | --- | --- | --- | --- | --- | --- | --- |
| **Public** | 7538 | 20.4 | 984 | 19.7 | 575 | 21.0 | 524 | 21.2 |
| **Social welfare corporation** | 17709 | 48.0 | 2405 | 48.1 | 1305 | 47.9 | 1171 | 47.4 |
| **Private companies** | 6725 | 18.2 | 917 | 18.5 | 351 | 12.9 | 320 | 13.0 |
| **Others** | 4947 | 13.4 | 691 | 13.8 | 461 | 16.9 | 422 | 17.0 |
| **missing** | - | - | 3 |  | 35 | 1.3 | 33 | 1.3 |
| **Total** | 36919*^2^ | 100.0 | 4997 | 100.0 | 2727 | 100.0 | 2470 | 100.0 |

^*1*2^ National data were obtained from official statistics provided by the Ministry of Health, Labour and Welfare and the Children and Families Agency of Japan for fiscal year 2024（Reiwa 6）, and therefore the total number of facilities differs slightly between facility type and operating body classifications.
